# Comparing two post-resuscitation debriefing frameworks: a randomized cross-over simulation study

**DOI:** 10.1186/s41077-026-00460-9

**Published:** 2026-07-03

**Authors:** Frederik Marynen, Marco Garcia van Bijsterveld, Jill Van In, Jorg Roosen, Steffen Fieuws, Domien Vanhonacker, Walther NKA van Mook, Iwan C.C. van der Horst, Philippe Dewolf

**Affiliations:** 1 Department of Anesthesia, University Hospitals Leuven, Herestraat 49, Leuven, 3000 Belgium; 2 Department of Emergency Medicine, University Hospitals Leuven, Herestraat 49, Leuven, 3000 Belgium; 3 STEPS Skills Lab KULeuven, Herestraat 49, Leuven, 3000 Belgium; 4 Interuniversity Institute for Biostatistics and Statistical Bioinformatics, KU Leuven – University of Leuven, Leuven, 3000 Belgium; 5 Department of Anaesthesiology and Perioperative Medicine, Brussels University Hospital (UZBrussel), Laarbeeklaan 101, Jette, 1090 Belgium; 6 Department of Intensive Care Medicine, Maastricht University Hospital, P. Debyelaan 25, Maastricht, 6229 HX the Netherlands; 7 School of Health Professions Education, Maastricht University, Maastricht, the Netherlands; 8 Cardiovascular Research Institute Maastricht CARIM, Maastricht University, Maastricht, the Netherlands

**Keywords:** Post-resuscitation debriefing, Simulation, Cardiac arrest, Debriefing frameworks, DISCERN, Post-Code Pause (PCP)

## Abstract

**Background:**

Post-resuscitation debriefing (PRD) enhances individual and team performance in emergency care, thereby improving patient outcomes and provider well-being. Despite support from the European Resuscitation Council and the American Heart Association, the optimal PRD framework remains undefined. This study compares how team members experience PRD when conducted using either the DISCERN or Post-Code Pause (PCP) debriefing framework in pre-hospital cardiac arrest simulations.

**Methods:**

In a randomized cross-over study, 40 medical doctors participated in four advanced life support (ALS) simulation scenarios. Participants acted exclusively within their usual roles as team leaders, while the remaining team members performed standardized roles in accordance with the scenario scripts. Each participant experienced two DISCERN and two PCP debriefings, with the order randomized. The primary outcome was the total score on the Debriefing Experience Scale (DES), with item scores as secondary outcomes. Linear models with generalized estimating equations (GEE) were used to account for repeated measures when comparing the mean scores between the debriefing frameworks.

**Results:**

A total of 158 DES questionnaires were analyzed. Mean total DES scores were 4.15 (4.02; 4.28) for PCP and 4.16 (4.02; 4.29) for DISCERN (*p* = 0.93 for the comparison). These scores indicate a favorable debriefing experience for both frameworks. At the DES item level, no statistically significant differences were observed between frameworks, except for perceived physical comfort in the debriefing environment, which was rated significantly higher for DISCERN (DISCERN = 4.44 (4.27; 4.62), PCP = 4.13 (3.96; 4.30), p = < 0.0001).

**Conclusion:**

DISCERN and PCP provide similarly favorable debriefing experiences following simulated cardiac arrest scenarios, with no significant difference in overall DES scores. These findings suggest that both frameworks can be implemented without compromising perceived debriefing quality. Further research should include more professionally diverse participant samples and assess long-term debriefing outcomes.

**Trial registration:**

Clinical Trial Center UZ Leuven, S65846 September 2021.

**Supplementary Information:**

The online version contains supplementary material available at https://doi.org/10.1186/s41077-026-00460-9.

## Background

Emergency physicians use post-resuscitation debriefing (PRD) to review cardiac arrest events, fostering experiential learning through discussions of team and individual performances [1]. PRD has been linked to improved technical skills, better patient outcomes (e.g., CPR quality, return of spontaneous circulation, in-hospital survival), and enhanced non-technical skills such as teamwork, communication, and leadership [1–7]. Consequently, the European Resuscitation Council (ERC) and the American Heart Association (AHA) recommend PRD in their guidelines [2, 8]. Additionally, debriefing has been associated with reduced stress, anxiety, and burnout, as well as increased self-confidence among healthcare providers [9–13]. However, concerns about potential negative psychological effects during complex case discussions indicate that further research on provider mental health is needed [6, 14–16].

A recent systematic review identified six primary frameworks used in emergency settings [1]. All were found to be effective, but none were considered superior, emphasizing the need for further comparative research. This aligns with more recent research, including the systematic review by Phillips et al., which found that although several clinical debriefing tools exist, the available evidence does not clearly support one framework as superior [17].

The intention of post-resuscitation debriefings may vary depending on the nature of the clinical event and the needs of the team involved [18]. Kolbe et al. highlight that the purpose of a debriefing should be clearly defined, as intentions related to learning from events or managing strong emotions require different approaches [18]. Consequently, different debriefing frameworks may be better suited to specific intentions, as their structure and emphasis influence how learning and emotional reflection are facilitated. Some frameworks primarily focus on clinical performance and team processes, whereas others explicitly incorporate elements that allow participants to acknowledge emotional reactions and reflect on the experience of the event. Understanding these differences in perspective of its aim, is important when selecting a debriefing approach in resuscitation settings.

In summary, current literature suggests that the selection of a debriefing tool should be tailored to the specific context, including the objectives of the debriefing, the clinical setting, and local barriers. Rather than assuming the existence of a universally superior framework, a context-sensitive approach is recommended [1, 17].

Moreover, a recent systematic scoping review reported fragmented and inconclusive evidence regarding the effects of debriefing practices on healthcare workers’ emotional outcomes [19]. Finally, empirical research directly comparing how different post-resuscitation debriefing approaches influence healthcare professionals’ perceived debriefing experience remains scarce [1, 17].

At our institution, efforts are underway to further implement and optimize post-resuscitation debriefing practices. To inform this process, it is important to better understand how different debriefing approaches may influence the experience of team members participating in these debriefing sessions.

Therefore, this study aims to explore whether two different debriefing frameworks with different inherent intentions, namely a stronger focus on clinical learning versus emotional reflection, are associated with differences in the perceived debriefing experience among healthcare professionals.

To address this question, we compared the participants’ debriefing experience between two established post-resuscitation debriefing frameworks that represent these different debriefing intentions: Debriefing In Situ Conversation after Emergent Resuscitation Now (DISCERN) and Post-Code Pause (PCP) [15, 16]. DISCERN focuses on performance review and team learning, while PCP focuses more on emotional reflection (Supplementary Material 1). Despite this difference in emphasis, both frameworks incorporate elements of clinical learning and psychological support. To enable a direct comparison, both frameworks were applied to debrief identical simulation scenarios. Participants’ debriefing experiences were evaluated using the Debriefing Experience Scale (DES) [20].

We used simulation as a methodological platform for this research question, serving as a surrogate for real-world practice and allowing controlled approximation of the clinical experience of team-based resuscitation. Prehospital resuscitation scenarios were designed to closely replicate prehospital clinical environment, including team composition and roles reflecting those encountered in routine practice within our institution. This approach allowed multiple debriefings to be conducted within a relatively short time frame, while ensuring a high degree of standardisation across both scenarios and debriefings.

### Debriefing In Situ Conversation after Emergent Resuscitation Now (DISCERN)

The DISCERN framework was developed as a standardized tool for post-resuscitation debriefings [15]. These debriefings are conducted as verbal debriefings using the plus-delta method, a structured approach in which participants first identify positive aspects of the performance (“plus”) and then discuss areas for improvement (“delta”), promoting balanced reflection and targeted feedback. Furthermore, DISCERN has served as a foundation for the development of other debriefing frameworks [21, 22].

### Post-code pause (PCP)

The PCP debriefing process was designed to evaluate team performance and address the psychological needs of staff following cardiac arrest [16]. It begins with a 10–15 s moment of silence to honor the patient and recognize the team’s efforts, facilitating immediate emotional processing. Copeland et al. reported that this approach reduced lingering emotional responses within 24 h after the event [16].

### Debriefing experience scale (DES)

The Debriefing Experience Scale (DES) is a widely used instrument designed to evaluate participants’ experience of debriefing following simulation training [23–25]. It was originally developed through literature review, expert input, and psychometric testing, resulting in a 20-item scale encompassing four core domains of the debriefing processes: reflection on thoughts and feelings, learning and making connections, facilitator skill, and facilitator guidance [20].

The DES was initially validated in nursing students, demonstrating high internal consistency for the experience scale [20]. Subsequent validation studies of translated versions of the DES generally demonstrated good psychometric properties across different cultural contexts [26–29]. These studies were conducted in populations including nursing students, nurses, and broader healthcare student groups. Although the DES has not been specifically validated for the participant group of this study (physicians), it was considered suitable given that the instrument evaluates fundamental, profession-independent dimensions of reflection, learning, and perceived facilitator support that represent core components of debriefing processes across healthcare professions.

## Methods

### Study setting

The data presented in this paper were obtained in the context of a randomised controlled trial (RCT) conducted at the University Hospitals Leuven (Belgium), with data collection taking place in the first half of 2022 (manuscript in preparation, see Supplementary Material 2). This parent study investigated the impact of implementing an extracorporeal cardiopulmonary resuscitation (ECPR) protocol on resuscitation quality, with each participant completing four standardized cardiac arrest scenarios in a pre-hospital setting (Supplementary Material 3).

### Simulation scenarios

The detailed content of the simulation scenarios is provided in Supplementary Material 3. Scenarios were based on pre-hospital cardiac arrest situations and integrated spatial and organisational challenges (e.g. evacuation constraints). Also, the scenarios incorporated emotionally charged components, including distressed bystanders and cases with significant psychosocial context (e.g. auto-intoxication). Patient outcomes varied across scenarios: in 75% of cases, the patient remained in cardiac arrest, whereas in 25% return of spontaneous circulation was achieved at the end of the scenario. These outcomes were predefined in the scenario scripts and occurred independently of participants’ clinical performance. The scenarios were standardized regarding information provided to each participant and lasted precisely 15 min.

During the simulations, the resuscitation team was structured to reflect standard practice in the Belgian pre-hospital setting, consisting of one physician, one nurse-paramedic, and two ambulance operators. The physician role was consistently performed by the study participant, reflecting their routine clinical function. The other team members, including the nurse-paramedic and ambulance personnel, were played by medical students who performed standardized roles and followed role-specific scripts as defined in the scenario design. They were thoroughly briefed in advance on their roles as team members and had a clear understanding of the scenario scripts.

Simulations were conducted in a dedicated Skills Lab (STEPS Skills Center KU Leuven). A prehospital environment was herein recreated to mimic real-world conditions, including typical operational constraints such as suboptimal working space. Resuscitation was performed using a Resusci Anne QCPR manikin (Laerdal, Stavanger, Norway) combined with the REALITi Go patient monitor simulator (iSimulate, Fyshwick, ACT, Australia).

### Participants

The participants in this study were medical doctors who were either pursuing a recognized specialization in either emergency medicine, anesthesiology, or internal medicine, or had already graduated in one of these disciplines. Additionally, completion of an ALS course within the five years preceding the start of the study was part of the inclusion criteria of the parent study.

A formal a priori power analysis was not conducted. The sample size of 40 participants was determined based on practical constraints and feasibility in the parent study, resulting in the use of a convenience sample. This embedded debriefing study used the same participant sample as the parent study.

### Study design

**Fig. 1:**
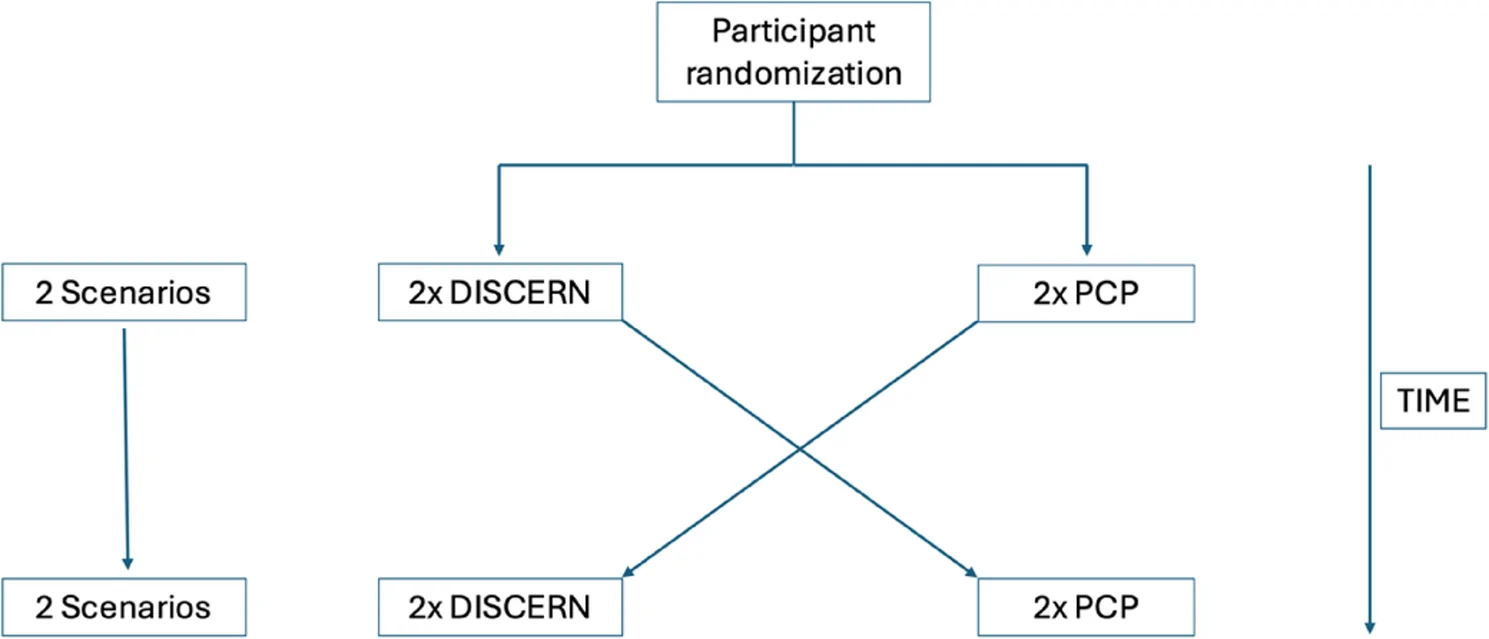
Cross-over design of the study. Participants completed four cardiac arrest simulations, receiving two DISCERN and two PCP debriefings in randomized order

By embedding a cross-over design within the parent study, the two debriefing frameworks were compared: each simulation was debriefed using either the DISCERN framework or the PCP framework (Fig. 1). Participants underwent two debriefings with each framework across their four simulations. The order in which participants received the DISCERN and PCP debriefing was determined based on the existing group allocation from the parent study (Supplemantary Material 2 and 4).

Each participant underwent two simulation sessions (corresponding to the two study phases of the parent study), each comprising two simulations and their corresponding debriefings, with a minimum interval of two weeks between sessions. Also, the study design ensured that every scenario was debriefed with both frameworks during both study phases, and in both the control and intervention groups of the parent study. This approach guaranteed balanced exposure to each debriefing method across all scenarios and study phases.

### Debriefing

The debriefings were conducted in the presence of the complete resuscitation team. Team members (medical students) were not provided with a script during the debriefing and encouraged to actively engage by sharing their perspectives and experiences from the simulation. Additionally, the debriefings were led by study investigators, all of whom were physicians with experience in simulation and advanced life support (ALS). The investigator who led a participant’s simulation also conducted their debriefing. A total of 6 investigators conducted the debriefings throughout the study, with each debriefer facilitating a different number of simulations. The allocation of debriefings to facilitators did not account for the type of debriefing framework used.

Prior to the study, all facilitators received extensive explanations and instructions on how to conduct both debriefing frameworks, provided by an expert in debriefing. Also, the study team participated in joint training sessions to ensure a consistent and effective approach to leading the debriefings and to become thoroughly familiar with both debriefing frameworks. During the study, each facilitator followed a scripted guide specific to the assigned framework, ensuring adherence to key elements and reducing variability in delivery.

### Evaluation instrument: debriefing experience scale (DES)

After each debriefing, participants completed the Debriefing Experience Scale (DES) to evaluate their perceptions of the debriefing process. This scale contains 20 items and uses a Likert-type rating (where 1= “Strongly disagree”, 2= “Disagree”, 3= “Undecided”, 4= “Agree”, and 5= “Strongly agree”), with an additional Not Applicable (NA) option. While the DES items are originally rated across two different areas (‘experience’ for the student and ‘importance’ of the experience to the student), only the experience part of the scale was used in this study.

### Outcomes

The primary outcome of the study was the total score on the DES questionnaire, calculated for each debriefing as the mean Likert score across all items, excluding those marked as “Not Applicable”. This total score was used to assess and compare the participants’ debriefing experience after application of both DISCERN and PCP debriefing.

The secondary outcome of this study was an item analysis of DES to identify specific areas where differences between frameworks may exist.

### Statistical methods

Descriptive information on the DES Likert scales is provided for each debriefing tool (PCP, DISCERN). Each participant contributes four observations (corresponding to the four simulations).

For each item separately, as well as for the total score, a linear model with generalized estimating equations (GEE) was used to handle the potential correlation between the four repeated measures per participant, and to compare the mean score between the two groups, correcting for the type of partcipants’ specialty (Emergency Medicine, Anesthesia or Internal Medicine) and scenario. Note that - apart from missing information - there is no imbalance in the distribution of the scenarios and specialties between both debriefing tools (since debriefing is a within-subject factor). Therefore, adding these terms to the model did not aim at removing bias, but only at decreasing the residual variance (and hence increasing the precision in the estimate for the difference between both tools). From the model, means (with 95% confidence interval) in both tools were reported, and the difference in mean score.

All analyses were performed using SAS software, version 9.4 of the SAS System for Windows.

### Language editing

Large Language Model (ChatGPT, OpenAI) was used in the preparation of this manuscript solely to improve readability and sentence structure, as the authors are not native English speakers. The authors reviewed and edited the text after this process and take full responsibility for the content of the manuscript.

## Results

### Participants and data exclusions

A total of 40 participants were initially recruited for this study. However, one participant completed only the pre-phase. Additionally, technical issues resulted in the loss of data recordings for two participants, leaving them with no usable data. Therefore, these two participants were excluded and were replaced with two newly recruited participants. These replacements were randomly assigned to the position of an excluded participant. This brings the total number of participants with data suitable for analysis to 40, with a total of 158 DES evaluations available for analysis. An overview of participant flow is provided in Supplementary Material 4.

### Primary outcome

**Fig. 2:**
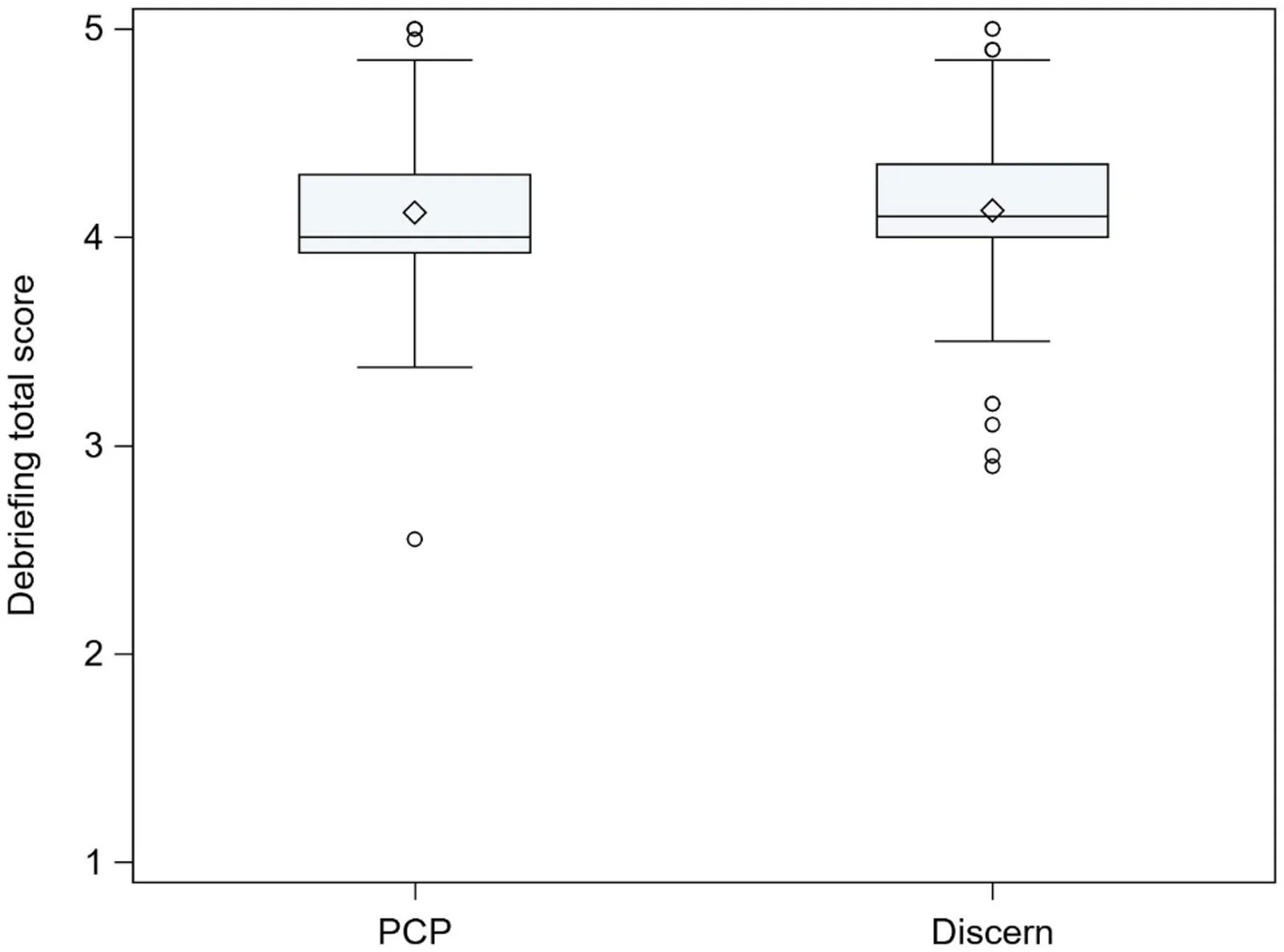
Debriefing total score comparison. Distribution of DES in PCP and DISCERN groups. The box corresponds to the interquartile range (IQR), whereas the whiskers extend to the most extreme values, being within 1.5 times the IQR from the box. Dots give values outside this range. The horizontal line and diamond symbol in the box are the median and mean value, respectively

The mean total DES score was similar (*p* = 0.93) for both debriefing methods (Table 1; Fig. 2). The mean total score for the PCP and DISCERN method was 4.15 (4.02; 4.28) and 4.16 (4.02; 4.29), respectively.

**Table 1:** Comparison of the total DES score between PCP and DISCERN

	Mean score (95%CI)			
	PCP	Discern	Difference (95%CI)	*P*-value
Total score	4.15 (4.02;4.28)	4.16 (4.02;4.29)	-0.00 (-0.09;0.08)	0.9341
Estimates from a linear model for repeated measures (using GEE)				

To calculate the total DES score, the mean Likert score was calculated over all items, excluding items marked as “Not Applicable”. The Likert scale used in the DES classifies participant responses as follows: 1 = “Strongly disagree,” 2 = “Disagree,” 3 = “Undecided,” 4 = “Agree,” and 5 = “Strongly agree.”

### Secondary outcome

The majority of the DES items showed generally favorable mean scores for both frameworks, with no statistically significant differences between them. Participants in both groups agreed that the debriefing helped them analyze their thoughts, connect their learning, reflect on their actions during the simulation, and that they benefited from the facilitator’s guidance throughout the debriefing (Table 2).

**Table 2:** Comparison of continuous Likert-scale scores between PCP and DISCERN for DES items

DES ITEMS	Mean score (95%CI)			
	PCP	Discern	Difference (95%CI)	*P*-value
Analyzing thoughts and feelings				
Debriefing helped me analyze my thoughts.	4.16 (4.02;4.31)	4.14 (3.99;4.29)	0.02 (-0.12;0.17)	0.7387
The facilitator (person who led the debrief) reviewed (discussed) aspects of the health care teams behavior.	4.30 (4.14;4.46)	4.27 (4.11;4.42)	0.03 (-0.13;0.20)	0.6752
The debriefing environment was physically comfortable.	4.13 (3.96;4.30)	4.44 (4.27;4.62)	-0.31 (-0.46;-0.17)	< 0.0001
Unsettled feelings from the simulation were resolved by debriefing.	4.04 (3.85;4.24)	4.07 (3.88;4.27)	-0.03 (-0.20;0.14)	0.7169
Learning and making connections				
Debriefing helped me make connections in my learning. For example, to connect past and present learning experiences.	4.19 (4.01;4.37)	4.19 (4.01;4.38)	-0.00 (-0.18;0.17)	0.9632
Debriefing was helpful in processing the simulation experience.	4.17 (4.03;4.32)	4.24 (4.10;4.39)	-0.07 (-0.21;0.08)	0.3455
Debriefing provided a learning opportunity.	4.23 (4.07;4.40)	4.34 (4.17;4.51)	-0.11 (-0.25;0.03)	0.1202
Debriefing helped me find meaning in the simulation.	4.01 (3.81;4.22)	3.94 (3.73;4.15)	0.08 (-0.11;0.26)	0.4068
My questions from the simulation were answered by debriefing.	4.05 (3.84;4.26)	4.01 (3.80;4.22)	0.04 (-0.16;0.24)	0.6836
I became more aware of myself during the debriefing session.	4.17 (3.99;4.35)	4.15 (3.97;4.34)	0.02 (-0.16;0.19)	0.8546
Debriefing helped me clarify problems.	4.19 (4.00;4.37)	4.15 (3.96;4.34)	0.04 (-0.11;0.18)	0.6334
Debriefing helped me make connections between theory and real-life situations.	4.05 (3.87;4.22)	3.98 (3.80;4.17)	0.06 (-0.14;0.26)	0.5238
Facilitator skills in conducting the debriefing				
The facilitator (person who led the debrief) allowed me enough time to verbalize my feelings before commenting.	4.28 (4.10;4.47)	4.28 (4.10;4.47)	0.00 (-0.16;0.17)	0.9809
The debriefing session facilitator (person who led the debrief) talked the right amount during debriefing.	4.28 (4.11;4.45)	4.21 (4.04;4.38)	0.08 (-0.11;0.26)	0.4073
Debriefing provided a means for me to reflect on my actions during the simulation.	4.28 (4.11;4.44)	4.29 (4.12;4.46)	-0.02 (-0.16;0.13)	0.8168
I had enough time to debrief thoroughly.	4.09 (3.85;4.32)	4.10 (3.86;4.33)	-0.01 (-0.20;0.18)	0.8996
The debriefing session facilitator (person who led the debrief) was an expert in the content area.	4.09 (3.86;4.31)	4.09 (3.86;4.32)	-0.00 (-0.16;0.16)	0.9950
Appropriate facilitator guidance				
The facilitator (person who led the debrief) taught the right amount during the debriefing session.	4.03 (3.80;4.26)	3.89 (3.66;4.12)	0.14 (-0.03;0.32)	0.1056
The facilitator (person who led the debrief) provided constructive evaluation of the simulation during debriefing.	4.10 (3.87;4.32)	4.11 (3.88;4.34)	-0.01 (-0.18;0.16)	0.8715
The facilitator (person who led the debrief) provided adequate guidance during the debriefing.	4.26 (4.08;4.44)	4.30 (4.12;4.49)	-0.04 (-0.20;0.11)	0.5629
Estimates from a linear model for repeated measures (using GEE)				

The Likert scale: 1= “Strongly disagree”, 2= “Disagree”, 3= “Undecided”, 4= “Agree”, and 5= “Strongly agree”

Only one item showed a statistically significant difference between the frameworks (Table 2). For the item “The debriefing environment was physically comfortable” the DISCERN framework achieved a significantly higher mean score compared to PCP (DISCERN = 4.44, PCP = 4.13, *p* < 0.0001). Under the DISCERN framework, in 47.4% of the evaluated debriefings, “completely agree” was selected for this item, compared to 23.8% under PCP. Additionally, in 51.3% of evaluated debriefings under DISCERN “agree” was selected, compared to 66.3% under PCP. An “undecided” response was selected in 1.3% evaluations under DISCERN, compared to 8.8% under PCP. Finally, “disagree” was not selected in any DISCERN evaluation, whereas under PCP, 1.3% of evaluations received this rating for this item (Supplementary Material 5).

In conclusion, both frameworks demonstrated similar overall effectiveness, as reflected in their favorable total DES scores. Significantly higher scores were observed in the DISCERN group for the item “The debriefing environment was physically comfortable”.

## Discussion

This study compared participants’ debriefing experiences across two post-resuscitation debriefing frameworks with different underlying intentions: DISCERN, which focuses on clinical performance and learning objectives, and PCP, which emphasizes emotional support and team reflection. The results of this study showed no significant difference in total DES scores between the two frameworks, suggesting that both debriefing approaches result in a comparable participant-reported debriefing experience.

Interestingly, there was a notable difference in responses to the DES item regarding the physical comfort of the debriefing environment. The DISCERN group scored significantly better on this item than the PCP group, despite the debriefings having occurred in the same physical space for both groups. The DES was developed to assess learners’ subjective experience of debriefing, rather than objective environmental factors [20]. As such, items such as “physical comfort” may also reflect broader aspects of the learning climate, including psychological safety and ease of participation. A possible explanation for the lower rating of perceived physical comfort in the PCP framework could be the presence of a 10-second silent pause or the psychologically oriented content of the session. While such factors might have influenced participants’ perceptions, this association remains speculative. Further qualitative studies are warranted to explore participants’ subjective experiences in more depth and to understand better the emotional impact of specific debriefing elements. However, given this observed difference, debriefing facilitators should pay extra attention to the physical comfort of participants when using the PCP model.

A prior mixed-methods study by Kam et al. compared DISCERN and PCP in 114 in situ debriefings following pediatric critical events and found both frameworks effective in identifying improvement points and supporting team reflection, with PCP being preferred for both emotional support and clinical education [30]. In our study, both debriefing frameworks received similar total DES scores, and only one DES item showed a statistically significant difference: DISCERN was rated higher in terms of perceived physical comfort. A possible explanation for the discrepancy in findings with Kam et al. lies in the differing clinical contexts. Their study involved real paediatric critical events, which are often highly emotionally charged, potentially allowing the strengths of PCP in addressing emotional needs to become more evident. In our simulation-based setting, the emotional intensity is inherently lower, which may have limited the extent to which such differences between frameworks could be observed.

The originality of our study lies in its randomized cross-over design with standardized simulations, allowing each participant to experience both frameworks under nearly identical conditions. This contrasts with prior work, such as the study by Kam et al., which evaluated frameworks in situ but lacked a within-subject comparison [30]. By isolating the influence of the framework itself from case-related variability, we demonstrate that the frameworks are largely interchangeable in terms of learner experience.

Our findings have direct implications for the clinical implementation of post-resuscitation debriefings. Both DISCERN and PCP yielded equally favorable debriefing experiences, reinforcing their suitability as structured approaches in daily practice. Given the strong endorsement of debriefing by the ERC and AHA, our results provide practical reassurance to educators and clinical leaders that either framework can be safely integrated without compromising the quality of the debriefing.

Although effective debriefing frameworks are available, their availibility and use alone do not guarantee systematic and optimal implementation of post-resuscitation debriefing. Several barriers may hinder this process, including time constraints, the need for a clear definition of the debriefing’s purpose, the requirement for adequate debriefing skills, and the establishment of a culture in which debriefing is normalized and supported, as described by Petrosoniak et al. [31].

Furthermore, the choice for a specific debriefing framework may be context-dependent and guided by the intended focus of the debriefing. Kolbe et al. emphasize that the intention of a debriefing should first be identified, after which the debriefing approach should be selected to align with that intention [18]. In contrast, our study explored this relationship in the opposite direction: two debriefing frameworks with different emphases and intentions (DISCERN focusing more on learning and clinical performance review and PCP incorporating elements for emotional reflection) were applied to debrief identical simulation scenarios. Despite these differences in underlying debriefing intentions, both frameworks resulted in similar debriefing experiences, as reflected by comparable total DES scores.

Future research could explore whether explicitly aligning the debriefing framework with predefined debriefing intentions influences learning or psychological outcomes. Also, further research is needed to assess the long-term learning and psychological effects of these debriefing frameworks.

### Limitations

No a priori sample size calculation was performed for this study. However, with the observed variance of the total score and the covariance between the repeated measures, there was with the included number of observations > 95% power to detect a difference of 0.2 points in total DES score. Nevertheless, the absence of a pre-specified sample size calculation remains a methodological limitation.

Also, the unequal distribution of debriefings across the six facilitators, combined with the fact that their allocation did not account for the type of debriefing framework used, represents a limitation of this study. Although all facilitators were thoroughly trained in both frameworks, participated in joint training sessions, and followed framework-specific scripted guides to promote consistency, facilitator-related factors may still have influenced participants’ debriefing experience. Therefore, the results of this study should be interpreted with caution.

Participant debriefing experience was selected as the primary outcome, as it represents a key component of the debriefing process and can be readily assessed immediately after the debriefing. However, other relevant outcomes of post-resuscitation debriefing, such as process improvement and patient outcomes, were not directly evaluated.

Another limitation relates to the use of simulation as a surrogate for real clinical practice. Although the simulation scenarios were designed to resemble real-world prehospital cardiac arrest situations, including the incorporation of emotionally charged elements, they cannot fully replicate the complexity, unpredictability, and emotional intensity of authentic clinical encounters. In real-life resuscitations, healthcare professionals are confronted with genuine patient outcomes, responsibility, and exposure to human suffering, which may elicit stronger and more diverse emotional responses than those experienced in a simulated setting. As a result, the dynamics of post-event debriefings in clinical practice may differ from those observed in this study. In particular, emotional reflection may play a more prominent role in real-world debriefings compared to simulation-based debriefings.This limits the generalizability of our findings to real clinical debriefings.

Additionally, since team members (medical students) followed standardized scenario scripts to ensure consistency across simulations, this inevitably reduced the authenticity of team dynamics within the resuscitation team. This may have influenced the discussion of team dynamics during debriefing and further limits the generalizability of our findings to real-world practice.

Furthermore, the use of the Debriefing Experience Scale (DES) represents an important contextual limitation. As the DES specifically relates to simulation debriefing experiences, it inherently reflects the educational focus of simulation training, placing greater emphasis on learning processes. This may lead to underrepresentation of items capturing nuanced emotional reflection, which may be more relevant in real clinical debriefings. Consequently, caution is warranted when applying these findings beyond the simulation context. In addition, the DES has not been specifically validated in physicians, which may limit the interpretability of our findings in this population.

Moreover, although the simulations involved complete multidisciplinary resuscitation teams in accordance with Belgian pre-hospital practice, only physicians were enrolled as formal participants and completed the debriefing evaluations. This limits the generalizability of the findings to other team members, such as paramedics or ambulance operators. Future studies should consider including a broader range of healthcare professionals to capture the debriefing experience across all roles involved in resuscitation.

Finally, participants were aware that two debriefing approaches were being compared, which may have introduced some response bias in their DES ratings.

## Conclusion

This study demonstrates that both the DISCERN and Post-Code Pause (PCP) debriefing frameworks provide a similarly favorable debriefing experience for physicians following identical cardiac arrest simulations. Despite their different focus on clinical performance and emotional reflection, no significant difference was observed in total Debriefing Experience Scale (DES) scores.

These findings suggest that both frameworks can be effectively implemented in practice without compromising the perceived quality of debriefing. Rather than favoring one framework over the other, the selection of a debriefing approach may be guided by the intended focus of specific debriefing sessions.

Future research should focus on more professionally diverse participant samples and incorporate long-term follow-up to better understand the broader impact of these frameworks.

## Supplementary Information

Supplementary Material 1.

Supplementary Material 2.

Supplementary Material 3.

Supplementary Material 4.

Supplementary Material 5.

## Data Availability

Data supporting the findings of this study are available from the corresponding author upon reasonable request.

## Abbreviations

ALS: Advanced Life Support
AHA: American Heart Association
DES: Debriefing Experience Scale
DISCERN: Debriefing In Situ Conversation after Emergent Resuscitation Now
ECPR: Extracorporeal Cardiopulmonary Resuscitation
ERC: European Resuscitation Council
GEE: Generalized Estimating Equations
MD: Medical Doctor
PCP: Post-Code Pause
PRD: Post-Resuscitation Debriefing
