# Supplementary material for: Comparing two post-resuscitation debriefing frameworks: a randomized cross-over simulation study
Source: Adv Simul (Lond). 2026 Jul 3;11:65. doi: 10.1186/s41077-026-00460-9 (PMC13599248; doi:10.1186/s41077-026-00460-9)
Supplement: Supplementary file 1 [file 41077_2026_460_MOESM4_ESM.pdf]

Integrated debriefing study design in the ECPR trial

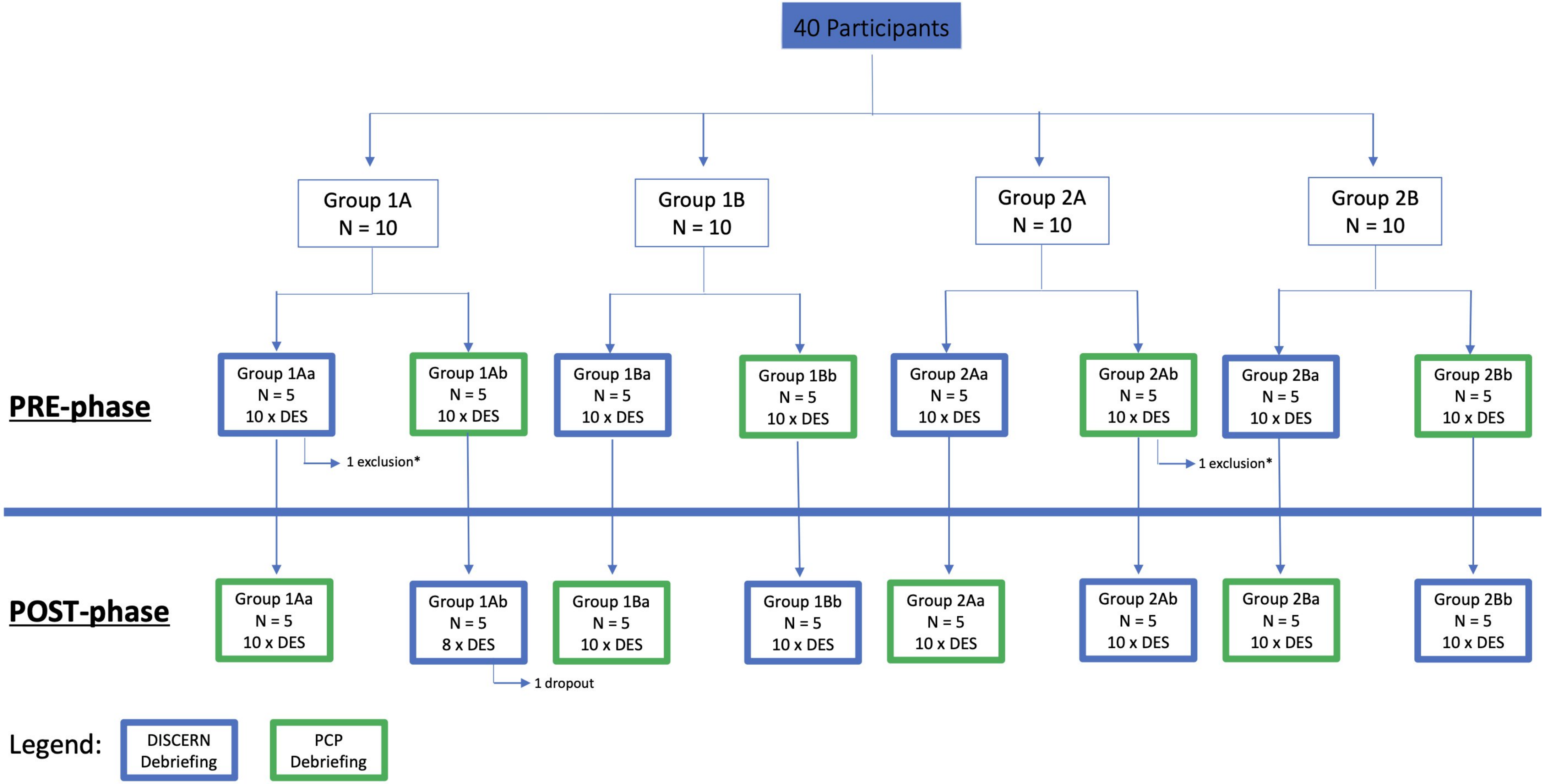

DES = Debriefing Experience Scale

* Excluded participants: 2 participants were excluded from the study due to technical failure during the pre-phase. These participants were replaced by two new participants.
