# Supplementary material for: Comparing two post-resuscitation debriefing frameworks: a randomized cross-over simulation study
Source: Adv Simul (Lond). 2026 Jul 3;11:65. doi: 10.1186/s41077-026-00460-9 (PMC13599248; doi:10.1186/s41077-026-00460-9)
Supplement: Supplementary file 2 [file 41077_2026_460_MOESM1_ESM.docx]

## Key structural distinctions between PCP and DISCERN debriefing frameworks

| **Aspect** | **PCP (Post-Code Pause)** | **DISCERN** |
| --- | --- | --- |
| Structure | Set of 7 scripted reflective questions, preceded by a 10–15 second pause | Based on plus-delta model with open prompts for strengths and potential improvements |
| Focus | Emphasizes emotional impact and psychological support | Emphasizes clinical performance and learning objectives |
| Emotional Components | Includes direct questions on well-being and readiness to return to work | Includes a closing statement regarding the availability of counseling support |
