# Supplementary material for: Comparing two post-resuscitation debriefing frameworks: a randomized cross-over simulation study
Source: Adv Simul (Lond). 2026 Jul 3;11:65. doi: 10.1186/s41077-026-00460-9 (PMC13599248; doi:10.1186/s41077-026-00460-9)
Supplement: Supplementary file 3 [file 41077_2026_460_MOESM2_ESM.docx]

**Study Design of the Parent ECPR Simulation Trial (Unpublished)**

The present debriefing study was embedded within a larger, currently unpublished randomized controlled simulation trial evaluating the impact of implementing an Extracorporeal Cardiopulmonary Resuscitation (ECPR) protocol on prehospital resuscitation quality. This parent trial was conducted in 2022 at University Hospitals Leuven and its design served as the structural foundation for the debriefing study presented in this manuscript.

**Study Design and Group Allocation**

Each participant completed four standardized cardiac arrest scenarios: two in the pre-phase (before implementation) and two in the post-phase (after implementation of the ECPR protocol for the intervention group). A minimum time interval of 14 days was maintained between pre-phase and post-phase.

In this ECPR trial 40 participants were initially randomized into four groups (1A, 1B, 2A, 2B) using permuted blocks with a size of 8.

- **Groups 1A and 1B** served as the **control group**, using the standard Advanced Life Support (ALS) protocol throughout both phases.
- **Groups 2A and 2B** formed the **intervention group**. During the pre-phase, they managed the scenarios using the standard Advanced Life Support (ALS) protocol, identical to the control groups. Between the pre- and post-phase, they completed a video course introducing the ECPR protocol. In the post-phase, they subsequently applied the newly introduced ECPR protocol during the simulation scenarios.

For the purpose of the debriefing study, each of these four groups (1A, 1B, 2A, 2B) were further subdivided into **2 subgroups (1Aa, 1Ab, 1Ba, 1Bb, 2Aa, 2Ab, 2Ba, 2Bb)** using permuted blocks with a size of 10. This subdivision ensured that every scenario was debriefed with both frameworks during both the pre- and post-phases, and in both the control and intervention groups of the ECPR study. This approach guaranteed balanced exposure to each debriefing method across all scenarios and study phases.
A detailed overview of this study design is provided in Additional File 4.

Both allocation sequences (the initial randomization into 4 groups and the subsequent subdivision into debriefing conditions) were created using a web-based randomization system (sealedenvelope.com), which was accessed by the study coordinator only after the participant was enrolled.

**ECPR Protocol Overview**

The ECPR protocol implemented in the intervention arm was based on Belgian prehospital practice and emphasized:

- Early recognition of ECPR candidates using predefined inclusion/exclusion criteria
- Notification of the receiving ECMO center within 10 minutes of arrival on scene
- Rapid hospital transport with continuous ALS, avoiding unnecessary on-scene delays, aiming for in-hospital cannulation as soon as possible

**Integration of the Debriefing Study**

The debriefing study followed a randomized cross-over design embedded within the larger study design. Each of the four simulations per participant was debriefed using either the DISCERN or Post-Code Pause (PCP) framework. The order was randomized and balanced through the eight-subgroup design, ensuring that:

- Each participant experienced two DISCERN and two PCP debriefings
- Each scenario was debriefed with both frameworks during both the pre- and post-phases, and in both the control and intervention groups of the ECPR study

After each debriefing, participants completed the Debriefing Experience Scale (DES) questionnaire to evaluate their subjective experience.

**Ethical Approval**

Both the ECPR trial and the embedded debriefing study were conducted under a single ethical protocol, approved by the Ethics Committee Research of University Hospitals Leuven (reference number S65846).
