# Supplementary material for: Comparing two post-resuscitation debriefing frameworks: a randomized cross-over simulation study
Source: Adv Simul (Lond). 2026 Jul 3;11:65. doi: 10.1186/s41077-026-00460-9 (PMC13599248; doi:10.1186/s41077-026-00460-9)
Supplement: Supplementary file 4 [file 41077_2026_460_MOESM3_ESM.docx]

**Scenarios**

**Simulation Protocol**

Before the start of the scenarios, participants were thoroughly briefed on how the simulation would be conducted. They were instructed to perform all actions as realistically as possible, respecting the natural timing and procedures of clinical practice.
Participants were also instructed to assume the role of team leader in the scenario, acting as the pre-hospital physician in charge of the resuscitation team.

Any information that could not be obtained due to the limitations of the simulation environment (e.g., diagnostic results, patient responses,..) were communicated directly by the scenario director upon request or following the completion of a related action. Additionally, both the resuscitation team (excluding the participant acting as team leader) and the bystanders were played by medical students who were thoroughly familiar with the scenario script.

To further enhance realism and consistency, predefined time durations were established for specific medical procedures. These durations are outlined in the table below and were to be respected during the scenarios. The resuscitation team was familiar with these timings and consistently respected them throughout the simulation scenarios.

| **Intervention** | **Duration** |
| --- | --- |
| Establish intravenous (IV) access | 1.5 minutes |
| Attempt and fail IV access (if specified in the scenario) | 1 minute (includes time to report failure to team leader) |
| Intraosseous (IO) access (bone drill) | 1 minute |
| Prepare medication | 30 seconds |
| Administer medication | 5 seconds + 10 seconds holding the limb up |
| Prepare and administer medication | 35 seconds + 10 seconds holding the limb up |
| Prepare intubation equipment (+ actively ask for tube size) | 30 seconds |
| Prepare equipment for laryngeal mask placement (+ actively ask for size) | 30 seconds |
| Applying capnography | 20 seconds |
| Call the fire department | 30 seconds |
| Measure blood glucose | 30 seconds |
| Measure temperature | 30 seconds |

**Scenario 1: hyperkalemia**

**Scene**

The patient is lying on their back against a wall. A bystander is performing CPR on the patient

**Participant briefing**

- Situation: You are on pre-hospital duty and receive an emergency dispatch call from the 112 control center
- Background: A 25-year-old man collapsed in the street
- Assessment: No further information about his medical condition
- Recommendation: BLS (phone-assisted CPR) is being performed by bystanders without AED. You arrive simultaneously with the ambulance, 7 minutes after the collapse

The participant is asked to repeat the briefing.

**Medical and technical information for scenario guidance**

**1. Cardiac arrest rhythm and timeline cues**

- Rhythm: Ventricular Fibrillation (VF) throughout the scenario
  - Only in pre-phase: ROSC occurs at the end of the scenario, accompanied with significant capnography raise.
- Minute 9: spouse of patient arrives

**2. Differential diagnosis – 4 Hs and 4 Ts**

• Hypoxia: No signs of peripheral or central cyanosis

• Hypovolemia: No supporting evidence

• Hypothermia: Outside temperature is 16°C

• Hypo-/Hyperkalemia:

o If hyperkalemia is considered by the participant, the ambulance operator will inform the participant that a dialysis fistula is visible on the contralateral arm to the IV access

o If the participant fully exposes the patient and actively searches for clinical signs, the scenario director will state that a dialysis fistula is visible

o If hyperkalemia is not mentioned by the participant, the instructor prompts an ambulance operator to report the presence of a dialysis fistula after 45 seconds of continuous reasoning by the participant about the etiology of the cardiac arrest

• Toxins: No supporting evidence

• Tension pneumothorax: Equal breath sounds bilaterally

• Tamponade: No prehospital echocardiography (TTE) available

• Thromboembolism:

o Calves are soft and non-tender

**3. Clinical parameters and observations**

- Pupil response:
  - Before adrenaline: isocoric, normal diameter, reactive to light
  - After adrenaline: isocoric, mydriatic, slightly reactive bilaterally
- Core temperature: 36°C
- Blood glucose: 180 mg/dL

**Scenario script – resuscitation team and bystanders**

1. Bystander interaction
   • A bystander is performing CPR at the time of arrival.
   • If the participant asks the bystander what happened, the following information is shared:
   "I was walking toward this young man when he suddenly collapsed. He had been walking normally. I called 112 immediately and started CPR with instructions over the phone."
2. Spouse interaction
   Minute 9: The spouse arrives on scene visibly distressed, in panic and hyperventilating.
   She immediately approaches the resuscitation area and interferes physically: touches the patient’s face and chest, pulls at the endotracheal tube, and asks panicked questions.

If the team leader does not stop the spouse from disrupting the resuscitation within 20 seconds:

- She begins actively disrupting CPR (e.g., obstructing chest compressions, interrupting rhythm checks)

If the team leader intervenes between minute 9 and 12 by firmly but respectfully preventing further disruption, and ensures the spouse is guided to a physical distance from the resuscitation scene:

- She allows herself to be guided to a distance from the patient, though remains emotional and anxious
- At this point she no longer disrupts the resuscitation
- In conversation, she remains distressed but provides clear answers when directly questioned by the team leader:
  - He has a kidney condition, "something with cysts."
  - He recently started dialysis but is awaiting a kidney transplant.
  - He skipped dialysis yesterday because he was tired of it.
  - His name is Jonas and he is 25 years old.
- She stays on scene for the remainder of the scenario as a calm bystander.

If the team leader does not intervene before minute 12:

- The spouse continues to disrupt high-quality resuscitation until minute 12.
- At minute 12, she suddenly leaves the scene in distress.
- If the participant tries to talk to her afterwards, they are told she is no longer visible.
- If a team member is sent to find her, they return after one minute stating they couldn't locate her.

Alternative intervention by team leader:

- If the team leader delegates the task to a team member to isolate the spouse, this happens smoothly.
- However, no questions about the medical history are asked unless the participant does so personally.

Behavior of resuscitation team during the disturbance:

- Team members continue their tasks to the best of their ability.
- They do not speak to the spouse nor move her unless specifically instructed by the team leader.

3. Evacuation
   • If the participant suggests transporting the patient before the end of the scenario, they are informed that a broken-down truck is blocking the only road to the hospital. The fire department has been notified to clear the path, but the blockage will not be resolved before the scenario ends.
   • It is also impossible to load the patient into the ambulance due to the traffic situation.

**Scenario 2: pulmonary embolism**

**Scene**

A female patient is receiving CPR from two ambulance operators

**Participant briefing**

- Situation: You are on pre-hospital duty and receive an emergency dispatch call from the 112 control center
- Background: The ambulance crew is requesting support for the sudden collapse of a young woman in her apartment. The initial 112 call reported mild shortness of breath. The apartment is located on the second floor and is only accessible via a narrow spiral staircase
- Assessment: No further information is available regarding the patient's medical history
- Recommendation: Upon arrival, the ambulance crew is performing CPR using a bag-valve mask and AED, 8 minutes after the collapse

The participant is asked to repeat the briefing.

**Medical and technical information for scenario guidance**

**1. Cardiac arrest rhythm and timeline cues**

- Rhythm: Pulseless Electrical Activity (PEA) throughout the scenario
- Minute 5 and 11: BLS provider shows fatigue, resulting in poor-quality chest compressions and a drop in capnography
- Minute 9: Ambulance operator mentions the younger sister is still in the apartment (only if participant has not yet inquired about bystanders)

**2. Differential diagnosis – 4 Hs and 4 Ts**

- Hypoxia: No signs of peripheral or central cyanosis
- Hypovolemia: Well-filled circulation, jugular veins distended, no signs of bleeding
- Hypothermia: Normal ambient temperature in the apartment
- Hypo-/Hyperkalemia: No information available
- Toxins: No signs of toxins near patient
- Tension pneumothorax: Equal breath sounds bilaterally
- Tamponade: No prehospital echocardiography (TTE) available
- Thromboembolism:
  - If thromboembolism is mentioned explicitly by the participant, the nurse paramedic says:
    "The right lower leg appears swollen and tense."
  - If the participant fully exposes the patient and actively searches for signs, the scenario director says:
    "You observe that the right lower leg is significantly swollen and tense."

**3. Clinical parameters and observations**

- Pupil response:
  - Before adrenaline: isocoric, normal diameter, reactive to light
  - After adrenaline: isocoric, mydriatic, slightly reactive bilaterally
- Core temperature: 36°C
- Blood glucose: 180 mg/dL
- Thrombolytics: Not available with the prehospital team

**Scenario script – resuscitation team and bystanders**

**Ambulance operator briefing to participant** (if participant actively inquires):
"When we arrived, the patient was conscious and very short of breath. While we were trying to measure her oxygen saturation, she suddenly became unresponsive. No breathing or pulse – we moved her from the chair to the floor and started resuscitation. AED advised now shocks."
"Her younger sister is also in the apartment but ran off when we started CPR, probably still somewhere in the apartment. She didn’t mention any known medical issues — we didn’t explicitly ask either because everything happened so quickly."
No active suggestion is made to go find the sister.

**Minute 9 (if the younger sister has not yet appeared)
Ambulance operator:** "Her younger sister is also in the apartment but ran off when we started CPR, probably still here somewhere…"

If the participant takes action to speak to the sister

- If a team member is sent: the sister returns with team member after 15 seconds
- If the participant goes personally: they engage in a conversation with the sister 15 seconds later at the scene

**Younger sister** (if approached by the participant):
"The patient is 32 years old, no known medical history, only takes oral contraceptives. She had ankle surgery a month ago and was supposed to inject herself to prevent clots, but I think she stopped doing that this past week. She’s been in bed all week.
This morning she had a lot of pain in her leg and was breathing more shallowly, so I called the ambulance."

If thromboembolism is mentioned aloud by participant:
**Nurse paramedic** says: "The right lower leg appears swollen and tense."

If the patient is fully exposed and examined:
**Scenario director** says: "You observe that the right lower leg is significantly swollen and tense."

If the participant considers evacuation **=>** Input from **the nurse paramedic**:

- Horizontal evacuation is necessary – cannot use the stairs and no elevator is available
- Suggestion to evacuate through the window with assistance from the fire department
- If the participant agrees, a team member contacts the 100-dispatch center to request fire department support
- The fire department will not arrive before the scenario ends

**Scenario 3: acute coronary syndrome**

**Scene**

Bystander CPR is being performed. A chair is positioned next to the patient’s left side at waist level.

**Participant briefing**

- Situation: You are on pre-hospital duty and receive an emergency dispatch call from the 112 control center
- **Background:** Collapse of a middle-aged football supporter next to the pitch.
- **Assessment:** No additional information about the patient’s medical history.
- **Recommendation:** Bystander BLS (phone-assisted CPR) without AED. You arrive simultaneously with the ambulance, 7 minutes after the collapse.

The participant is asked to repeat the briefing.

**Medical and technical information for scenario guidance**

**1. Cardiac arrest rhythm and timeline cues**

- Rhythm: Ventricular Fibrillation (VF) throughout the scenario
  - Only in pre-phase: ROSC occurs at the end of the scenario, accompanied with significant capnography raise.
- Minute 6: BLS provider shows fatigue, resulting in poor-quality chest compressions and a drop in capnography

**2. Differential diagnosis – 4 Hs and 4 Ts**

• Hypoxia: No signs of peripheral or central cyanosis

• Hypovolemia: No supporting evidence

• Hypothermia: Outside temperature is 18°C

• Hypo-/Hyperkalemia: No supporting evidence

• Toxins: No supporting evidence

• Tension pneumothorax: Equal breath sounds bilaterally

• Tamponade: No prehospital echocardiography (TTE) available

• Thromboembolism: calves are soft and non-tender

**3. Clinical parameters and observations**

- Pupil response:
  - Before adrenaline: isocoric, normal diameter, reactive to light
  - After adrenaline: isocoric, mydriatic, slightly reactive bilaterally
- Core temperature: 36°C
- Blood glucose: 180 mg/dL

**Scenario script – resuscitation team and bystanders**

Bystander Script:
Answers only upon direct questioning by the participant; calm, non-obstructive behavior.

- “Ludo is a smoker and takes pills for high cholesterol.”
- “Ludo is 54 years old.”
- “Ludo collapsed by the sidelines. He was getting worked up again because his team was losing.”
- “During the break in the canteen, Ludo mentioned he was having chest pain.”

If the team leader attempts to initiate transport to the hospital, this is not possible. A bus has broken down at the exit of the parking lot, fully blocking access (to be communicated by the simulation Director). Placing the patient into the ambulance is also impossible due to this obstruction — the bus prevents any passage to the ambulance. The fire department has been alerted and will work to clear access, but this will not be resolved before the end of the scenario.

**Scenario 4: amitriptyline overdose**

**Scene**

The daughter is performing bystander CPR on the patient.
The patient is lying on her back on the ground, with two chairs positioned around her: one at the side of the patient (the daughter is performing chest compressions from the opposite side), and one positioned close to the patient's head.

**Participant briefing**

- **Situation**: You are on pre-hospital duty and receive an emergency dispatch call from the 112 control center
- **Background:** A 50-year-old woman has collapsed in her apartment. The apartment is located on the third floor and is only accessible via a narrow spiral staircase
- **Assessment:** No further information regarding the patient’s medical condition
- **Recommendation:** Bystander CPR (Phone-CPR) is being performed by the daughter, without AED. You arrive simultaneously with the ambulance, 6 minutes after the collapse.

The participant is asked to repeat the briefing.

**Medical and technical information for scenario guidance**

**1. Cardiac arrest rhythm and timeline cues**

- Rhythm: pulseless Ventricual Tachycardia (VT) throughout the scenario
- IV access is unsuccesful at every attempt. IO access is succesful at first attempt.
- Minute 7: BLS provider shows fatigue, resulting in poor-quality chest compressions and a drop in capnography
- **Interval minute 8–10:** nurse-paramedic inquires about evacuation.
- **Minute 11:** Ambulance operator finds five empty strips of Amitriptyline.

**2. Differential diagnosis – 4 Hs and 4 Ts**

• Hypoxia: No signs of peripheral or central cyanosis

• Hypovolemia: No supporting evidence

• Hypothermia: Temperature in the room is 21°C

• Hypo-/Hyperkalemia: No supporting evidence

• Toxins: Upon specific questioning by the team leader, the paramedic mentions having seen many empty wine bottles in the kitchen upon entering. The empty strips of Amitriptyline are only discovered at minute 11—there is no trace of them before that point.

• Tension pneumothorax: Equal breath sounds bilaterally

• Tamponade: No prehospital echocardiography (TTE) available

• Thromboembolism: calves are soft and non-tender

**3. Clinical parameters and observations**

- Pupil response:
  - Before adrenaline: isocoric, normal diameter, reactive to light
  - After adrenaline: isocoric, mydriatic, slightly reactive bilaterally
- Core temperature: 36°C
- Blood glucose: 180 mg/dL

**Scenario script – resuscitation team and bystanders**

Daughter (on specific prompting by the team leader):

- “My mother just sent me a message saying she needed to talk and wanted to get something off her chest. She had another argument with her boyfriend—they're in a very unstable relationship right now. I live on the first floor and came upstairs a few minutes later. When I arrived, she was lying on the couch and unresponsive. I started chest compressions on instructions from the emergency dispatcher.”

Additional answers to specific questions from the team leader:

- Entered the apartment no more than 2 minutes after receiving the text message.
- The patient is 58 years old.
- The patient is seeing a psychiatrist for depression. She takes multiple medications, but the daughter doesn't know which ones. No other known medical history.
- The patient has never attempted suicide before.
- She has been feeling very down and depressed lately.
- The patient has been drinking a lot of alcohol recently, linked to her depressive symptoms.
- The apartment is always very messy.

Team member (at minute 11):

- “Hey, I just found some empty medication strips next to the couch—it says amitriptyline on them.”

Nurse-paramedic (input during a evacuation discussion):

- Horizontal evacuation is necessary; evacuation via stairs is not feasible—there is no elevator.
- Proposes horizontal evacuation through the window using the fire department.
- If the team leader agrees, a team member will contact the emergency dispatch center to request fire department assistance for horizontal evacuation.
- The fire department will not arrive before the end of the scenario.

Inquiry about evacuation by nurse-paramedic:

- Question:“Doctor, how do you plan to evacuate this patient to the ambulance?”
- Timing:
  This question must be posed between minute 8 and 10 of the scenario. It should be introduced as early as reasonably possible within this time window. However, it must not be asked during or immediately preceding rhythm analysis or shock delivery. Additionally, if the team leader is actively verbalizing the 4 H/T assessment at minute 8, the question should be withheld until the team leader finishes speaking or leaves a pause.

If the opportunity to ask the question has not yet arisen by minute 10, it should then be asked—even if the team leader is in the process of reasoning aloud—unless a rhythm analysis or shock delivery is occurring. In such cases, the question must be asked immediately after the shock is delivered.
