# Supplementary material for: Comparing two post-resuscitation debriefing frameworks: a randomized cross-over simulation study
Source: Adv Simul (Lond). 2026 Jul 3;11:65. doi: 10.1186/s41077-026-00460-9 (PMC13599248; doi:10.1186/s41077-026-00460-9)
Supplement: Supplementary file 5 [file 41077_2026_460_MOESM5_ESM.docx]

## **DES Likert scale categorical**

Categorical Likert scale results from the completed DES.

| *Variable* | *Statistic* | *PCP* | *Discern* |
| --- | --- | --- | --- |
| **Debriefing helped me analyze my thoughts.** |  |  |  |
| Disagree | n/N (%) | 1/80 ( 1.25%) | 0/78 ( 0.00%) |
| Undecided | n/N (%) | 4/80 ( 5.00%) | 6/78 ( 7.69%) |
| Agree | n/N (%) | 58/80 ( 72.50%) | 57/78 ( 73.08%) |
| Completely agree | n/N (%) | 17/80 ( 21.25%) | 15/78 ( 19.23%) |
| **The facilitator (person who led the debrief) reviewed (discussed) aspects of the health care teams behavior.** |  |  |  |
| Disagree | n/N (%) | 1/80 ( 1.25%) | 2/78 ( 2.56%) |
| Undecided | n/N (%) | 0/80 ( 0.00%) | 3/78 ( 3.85%) |
| Agree | n/N (%) | 56/80 ( 70.00%) | 48/78 ( 61.54%) |
| Completely agree | n/N (%) | 23/80 ( 28.75%) | 25/78 ( 32.05%) |
| **The debriefing environment was physically comfortable.** |  |  |  |
| Disagree | n/N (%) | 1/80 ( 1.25%) | 0/78 ( 0.00%) |
| Undecided | n/N (%) | 7/80 ( 8.75%) | 1/78 ( 1.28%) |
| Agree | n/N (%) | 53/80 ( 66.25%) | 40/78 ( 51.28%) |
| Completely agree | n/N (%) | 19/80 ( 23.75%) | 37/78 ( 47.44%) |
| **Unsettled feelings from the simulation were resolved by debriefing.** |  |  |  |
| Disagree | n/N (%) | 3/80 ( 3.75%) | 2/78 ( 2.56%) |
| Undecided | n/N (%) | 9/80 (11.25%) | 8/78 (10.26%) |
| Agree | n/N (%) | 50/80 (62.50%) | 49/78 (62.82%) |
| Completely agree  Not-applicable | n/N (%)  n/N (%) | 16/80 (20.00%)  2/80 (2.50%) | 17/78 (21.79%)  2/78 (2.56%) |
| **Debriefing helped me make connections in my learning. For example, to connect past and present learning experiences.** |  |  |  |
| Completely disagree | n/N (%) | 1/80 ( 1.25%) | 0/78 ( 0.00%) |
| Disagree | n/N (%) | 1/80 ( 1.25%) | 2/78 ( 2.56%) |
| Undecided | n/N (%) | 4/80 ( 5.00%) | 5/78 ( 6.41%) |
| Agree | n/N (%) | 53/80 ( 66.25%) | 50/78 ( 64.10%) |
| Completely agree | n/N (%) | 21/80 ( 26.25%) | 21/78 ( 26.92%) |
| **Debriefing was helpful in processing the simulation experience.** |  |  |  |
| Disagree | n/N (%) | 1/80 ( 1.25%) | 0/78 ( 0.00%) |
| Undecided | n/N (%) | 3/80 ( 3.75%) | 4/78 ( 5.13%) |
| Agree | n/N (%) | 60/80 ( 75.00%) | 54/78 ( 69.23%) |
| Completely agree | n/N (%) | 16/80 ( 20.00%) | 20/78 ( 25.64%) |
| **Debriefing provided a learning opportunity.** |  |  |  |
| Disagree | n/N (%) | 1/80 ( 1.25%) | 0/78 ( 0.00%) |
| Undecided | n/N (%) | 5/80 ( 6.25%) | 4/78 ( 5.13%) |
| Agree | n/N (%) | 52/80 ( 65.00%) | 48/78 ( 61.54%) |
| Completely agree  Not-applicable | n/N (%)  n/N (%) | 21/80 ( 26.25%)  1/80 ( 1.25%) | 26/78 ( 33.33%)  0/78 |
| **Debriefing helped me find meaning in the simulation.** |  |  |  |
| Completely disagree | n/N (%) | 1/80 ( 1.25%) | 2/78 ( 2.56%) |
| Disagree | n/N (%) | 1/80 ( 1.25%) | 2/78 ( 2.56%) |
| Undecided | n/N (%) | 9/80 ( 11.25%) | 11/78 ( 14.10%) |
| Agree | n/N (%) | 57/80 ( 71.25%) | 50/78 ( 64.10%) |
| Completely agree | n/N (%) | 12/80 ( 15.00%) | 13/78 ( 16.67%) |
| **My questions from the simulation were answered by debriefing.** |  |  |  |
| Completely disagree | n/N (%) | 1/80 ( 1.25 %) | 1/78 ( 1.28%) |
| Disagree | n/N (%) | 1/80 ( 1.25 %) | 3/78 ( 3.85%) |
| Undecided | n/N (%) | 14/80 ( 17.50%) | 10/78 ( 12.82%) |
| Agree | n/N (%) | 45/80 ( 56.25%) | 48/78 ( 61.53%) |
| Completely agree  Not-applicable | n/N (%)  n/N (%) | 18/80 ( 22.50%)  1/80 ( 1.25%) | 15/78 ( 19.23%)  1/78 ( 1.28%) |
| **I became more aware of myself during the debriefing session**. |  |  |  |
| Disagree | n/N (%) | 3/80 ( 3.75%) | 1/78 ( 1.28%) |
| Undecided | n/N (%) | 5/80 ( 6.25%) | 9/78 ( 11.54%) |
| Agree | n/N (%) | 50/80 ( 62.50%) | 49/78 ( 62.82%) |
| Completely agree  Not-applicable | n/N (%)  n/N (%) | 21/80 ( 26.25%)  1/80 ( 1.25%) | 19/78 ( 24.36%)  0/78 ( 0.00%) |
| **Debriefing helped me clarify problems.** |  |  |  |
| Disagree | n/N (%) | 2/80 ( 2.50%) | 2/78 ( 2.56%) |
| Undecided | n/N (%) | 4/80 ( 5.00%) | 8/78 ( 10.26%) |
| Agree | n/N (%) | 56/80 ( 70.00%) | 49/78 ( 62.82%) |
| Completely agree | n/N (%) | 18/80 ( 22.50%) | 19/78 ( 24.36%) |
| **Debriefing helped me make connections between theory and real-life situations.** |  |  |  |
| Completely disagree | n/N (%) | 0/80 ( 0.00%) | 1/78 ( 1.28%) |
| Disagree | n/N (%) | 3/80 ( 3.75%) | 2/78 ( 2.56%) |
| Undecided | n/N (%) | 6/80 ( 7.50%) | 13/78 ( 16.67%) |
| Agree | n/N (%) | 57/80 ( 71.25%) | 45/78 ( 57.69%) |
| Completely agree | n/N (%) | 14/80 ( 17.50%) | 17/78 ( 21.79%) |
| **The facilitator (person who led the debrief) allowed me enough time to verbalize my feelings before commenting.** |  |  |  |
| Disagree | n/N (%) | 1/80 ( 1.25%) | 3/78 ( 3.85%) |
| Undecided | n/N (%) | 2/80 ( 2.50%) | 3/78 ( 3.85%) |
| Agree | n/N (%) | 51/80 ( 63.75%) | 41/78 ( 52.56%) |
| Completely agree | n/N (%) | 26/80 ( 32.50%) | 31/78 ( 39.74%) |
| **The debriefing session facilitator (person who led the debrief) talked the right amount during debriefing.** |  |  |  |
| Disagree | n/N (%) | 0/80 ( 0.00%) | 4/78 ( 5.13%) |
| Undecided | n/N (%) | 2/80 ( 2.50%) | 3/78 ( 3.85%) |
| Agree | n/N (%) | 54/80 ( 67.50%) | 44/78 ( 56.41%) |
| Completely agree | n/N (%) | 24/80 ( 30.00%) | 27/78 ( 34.62%) |
| **Debriefing provided a means for me to reflect on my actions during the simulation.** |  |  |  |
| Disagree | n/N (%) | 1/80 ( 1.25%) | 0/78 ( 0.00%) |
| Undecided | n/N (%) | 3/80 ( 3.75%) | 4/78 ( 5.13%) |
| Agree | n/N (%) | 51/80 ( 63.75%) | 49/78 ( 62.82%) |
| Completely agree | n/N (%) | 25/80 ( 31.25%) | 25/78 ( 32.05%) |
| **I had enough time to debrief thoroughly.** |  |  |  |
| Completely disagree | n/N (%) | 0/80 ( 0.00%) | 2/78 ( 2.56%) |
| Disagree | n/N (%) | 4/80 ( 5.00%) | 4/78 ( 5.13%) |
| Undecided | n/N (%) | 5/80 ( 6.25%) | 2/78 ( 2.56%) |
| Agree | n/N (%) | 52/80 ( 65.00%) | 47/78 ( 60.26%) |
| Completely agree | n/N (%) | 19/80 ( 23.75%) | 23/78 ( 29.49%) |
| **The debriefing session facilitator (person who led the debrief) was an expert in the content area.** |  |  |  |
| Disagree | n/N (%) | 2/80 ( 2.50%) | 4/78 ( 5.13%) |
| Undecided | n/N (%) | 13/80 ( 16.25%) | 10/78 ( 12.82%) |
| Agree | n/N (%) | 45/80 ( 56.25%) | 43/78 ( 55.13%) |
| Completely agree | n/N (%) | 20/80 ( 25.00%) | 21/78 ( 26.92%) |
| **The facilitator (person who led the debrief) taught the right amount during the debriefing session.** |  |  |  |
| Completely disagree | n/N (%) | 1/80 ( 1.25%) | 2/78 ( 2.56%) |
| Disagree | n/N (%) | 3/80 ( 3.75%) | 4/78 ( 5.13%) |
| Undecided | n/N (%) | 7/80 ( 8.75%) | 10/78 ( 12.82%) |
| Agree | n/N (%) | 53/80 ( 66.25%) | 49/78 ( 62.82%) |
| Completely agree | n/N (%) | 16/80 ( 20.00%) | 13/78 ( 16.67%) |
| **The facilitator (person who led the debrief) provided constructive evaluation of the simulation during the debriefing** |  |  |  |
| Completely disagree | n/N (%) | 0/80 ( 0.00%) | 1/78 ( 1.28%) |
| Disagree | n/N (%) | 5/80 ( 6.25%) | 4/78 ( 5.13%) |
| Undecided | n/N (%) | 6/80 ( 7.50%) | 3/78 ( 3.85%) |
| Agree | n/N (%) | 48/80 ( 60.00%) | 50/78 ( 64.10%) |
| Completely agree | n/N (%) | 21/80 ( 26.25%) | 20/78 ( 25.64%) |
| **The facilitator (person who led the debrief) provided adequate guidance during the debriefing.** |  |  |  |
| Disagree | n/N (%) | 1/80 ( 1.25%) | 3/78 ( 3.85%) |
| Undecided | n/N (%) | 4/80 ( 5.00%) | 2/78 ( 2.56%) |
| Agree | n/N (%) | 54/80 ( 67.50%) | 47/78 ( 60.26%) |
| Completely agree | n/N (%) | 21/80 ( 26.25%) | 26/78 ( 33.33%) |
